# The ECOLANG Multimodal Corpus of adult-child and adult-adult Language

**DOI:** 10.1038/s41597-025-04405-1

**Published:** 2025-01-16

**Authors:** Yan Gu, Ed Donnellan, Beata Grzyb, Gwen Brekelmans, Margherita Murgiano, Ricarda Brieke, Pamela Perniss, Gabriella Vigliocco

**Affiliations:** 1 Experimental Psychology, University College London, London, United Kingdom; 2 Department of Psychology, University of Essex, Colchester, United Kingdom; 3 Department of Psychology, University of Warwick, Coventry, United Kingdom; 4 Department of Biological and Experimental Psychology, Queen Mary University of London, London, United Kingdom; 5 Department of Rehabilitation and Special Education, University of Cologne, Cologne, Germany

**Keywords:** Human behaviour, Communication

## Abstract

Communication comprises a wealth of multimodal signals (e.g., gestures, eye gaze, intonation) in addition to speech and there is a growing interest in the study of multimodal language by psychologists, linguists, neuroscientists and computer scientists. The ECOLANG corpus provides audiovisual recordings and ELAN annotations of multimodal behaviours (speech transcription, gesture, object manipulation, and eye gaze) by British and American English-speaking adults engaged in semi-naturalistic conversation with their child (N = 38, children 3-4 years old, face-blurred) or a familiar adult (N = 31). Speakers were asked to talk about objects to their interlocutors. We further manipulated whether the objects were familiar or novel to the interlocutor and whether the objects could be seen and manipulated (present or absent) during the conversation. These conditions reflect common interaction scenarios in real-world communication. Thus, ECOLANG provides ecologically-valid data about the distribution and co-occurrence of multimodal signals across these conditions for cognitive scientists and neuroscientists interested in addressing questions concerning real-world language acquisition, production and comprehension, and for computer scientists to develop multimodal language models and more human-like artificial agents.

## Background & Summary

To account for the human capacity for language, we must study language in its core ecological niche, i.e., the real-world face-to-face settings that represent the conditions in which language has evolved, is learnt, and is commonly used. While most of the research concerning language processing, learning, and the neurobiology of language has focused on speech or text only, there is a long-standing tradition demonstrating the importance of information typically available in face-to-face communication, namely *multimodal signals*, such as prosody, gestures and face in language development, language production and comprehension^1–6^. In the real world, speakers produce multiple signals, both vocal and gestural at the same time and learners/listeners can dynamically weigh their importance. Recently, a growing number of studies have started to consider speech, gesture, prosody, and facial expression together^7–13^. However, to understand the cognitive and neural underpinning of real-world communication, and to develop more human-like artificial agents, we need first to know the distribution of the multiple cues in speakers’ production in different contexts resembling real-world communication. Some multimodal corpora have already been collected providing important resources for research^14–21^. However, given our interest in investigating the use of multimodal signals both in developmental and adult research, these previously collected corpora have important limitations. Among the open-access adult corpora, one of the largest available (CANDOR^14^) does not include any annotation of gestural behaviours (gestures are also more difficult to see in the videos from this corpus because it was collected via Zoom); another includes a majority of bi/multilingual individuals (MULTISIMO^16^) whose multimodal behaviours may be different from those of monolinguals. A lack of annotation for non-verbal behaviours is also the case for the CAAB dataset in which dyads are engaged in a referential communication game^22^. Other interesting corpora have been collected and annotated but are not publicly available. Examples include the Bielefeld Speech and Gesture Alignment (SaGA) Corpus comprising detailed annotations of both speech and gesture of adult German speakers providing route and landmark descriptions^23^ and the Nijmegen Corpus comprising recordings of one-hour-long conversations in Dutch on different topics by dyads of friends (e.g.^24^). Considering existing multimodal corpora of child-directed language^25^, although there is at least one very large longitudinal corpus (Goldin-Meadow’s^26^) we are not aware of any publicly available corpus except Tamis-LeMonda and Adolph’s^27^ dataset about young infants (13, 18 and 24 months), while there are a number of corpora annotated for speech only (see Homebank^28^). Thus, there is a need for the collection of a multimodal, annotated corpus of dyadic, face-to-face communication (including adult-to-adult and adult-to-child) in the different real-world settings in which communication occurs. The ECOLANG corpus addresses this gap, by providing an annotated corpus including speakers’ speech transcription, gesture and gaze annotation. Moreover, it is a resource that has the potential to be enriched with additional annotations, such as those for speech, prosody and potentially gestures of the addressee. It could also include annotations for additional multimodal cues, such as facial expressions of the speaker and adult addressee (excluding children, whose faces have been blurred), as well as other non-manual gestures.

When deciding how to go about collecting this corpus, we made several decisions. First of all, we wanted the corpus to be relevant to researchers working on development in children as well as researchers working on language production and comprehension in adults. Hence, we collected both dyadic communication between a caregiver and their child and between two familiar adults, thus introducing a manipulation of the type of addressee. Children included in the corpus are between 3 and 4 years of age. At this age children are still learning words at a fast pace and can acquire the use of more complex multimodal signals such as iconic (evoking properties of a referent talked about, e.g., drawing a circle in the air with a finger while talking about a ball) and metaphorical (imagistically linked to abstract concepts, e.g., a movement of the hand upward while talking about inflation) gestures^29–32^, as well as the pragmatic functions of prosody (e.g., raise in pitch at the beginning of a new topic of conversation)^33^. As the two parts of the corpus (adult-child and adult-adult) were collected in comparable conditions, this manipulation allows for the comparison between adults’ multimodal communication with children vs. other adults.

Second, we wanted the corpus to capture the real-world intertwining of episodes in which the conversants talk about something they both know, and cases in which one of the conversants is more knowledgeable. The latter is the typical situation studied in language acquisition studies where the caregiver is more knowledgeable than the child, but it is also a relatively common situation in adult social interactions (e.g., educational settings or just simply when another person tells us about something new)^34–37^. In the corpus, dyads converse about a provided set of objects where we manipulate their familiarity (i.e., whether the discussed object was familiar or unfamiliar to the interlocutor). Cases in which the addressee (child/adult) is unfamiliar with the object and its label provide learning opportunities, thus we can characterise how speakers use multimodal signals when teaching a new concept/label to a child or an adult. This is important because in the literature, typically, there is a sharp separation between studies addressing the role of caregivers’ multimodal behaviours in children’s learning^38–45^; *and* studies addressing the role of multimodal behaviours in adults’ comprehension and memory^2,10,45–51^. Thus, we do not know whether the multimodal cues provided by caregivers to their children are specifically associated with the cognitive development of the child, or to the learning context (but see^52^).

**Fig. 1:**
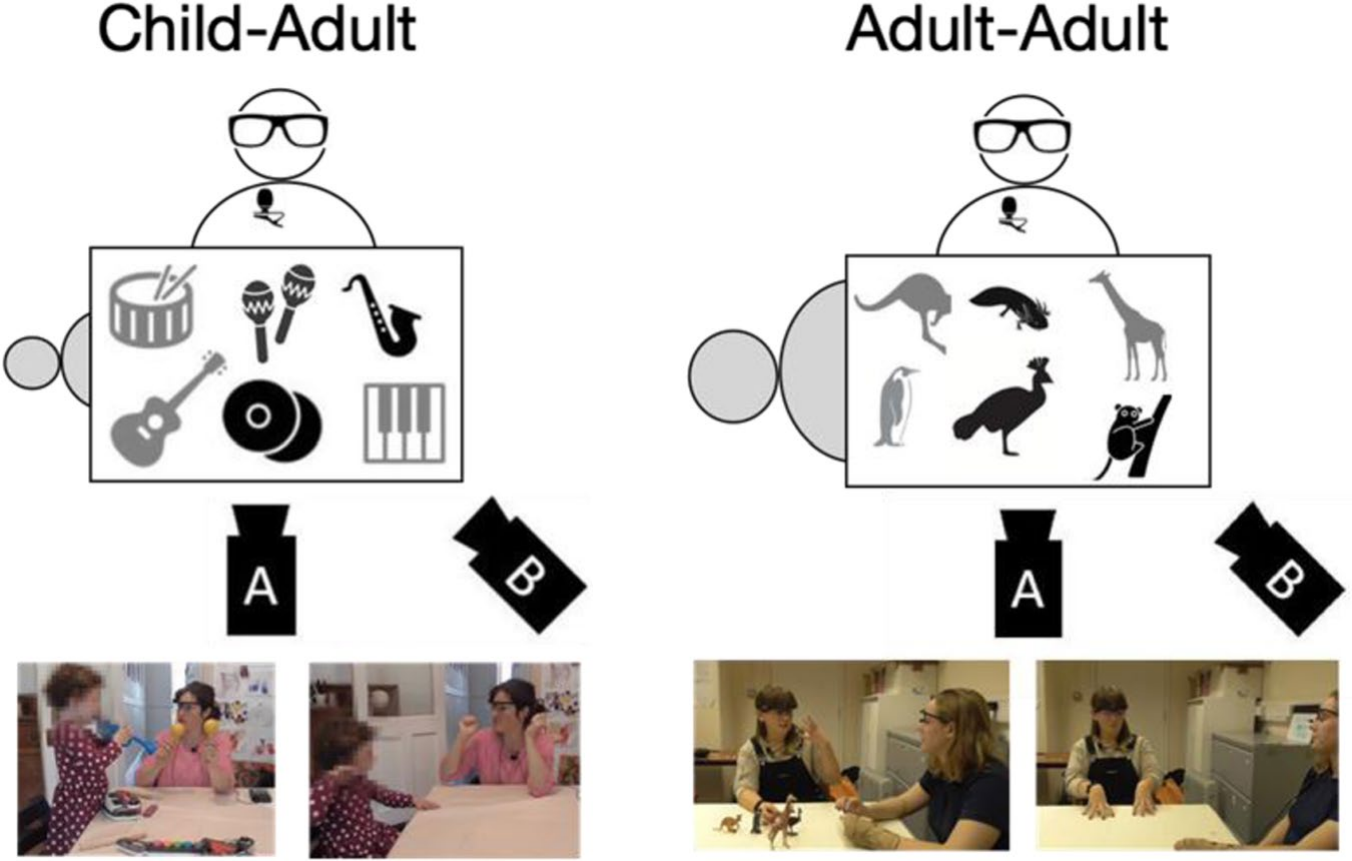
Corpus design. The top panel shows the recording setup. Speaker (white figure) and child/adult interlocutor (grey figure) sit at a table at 90 degrees to each other with objects from each category on the table. Two cameras record the interaction: camera A focuses on the speaker, camera B on the interaction space. The speaker wears Tobii eye-tracking glasses and a lapel microphone. Grey objects on the table are known to the interlocutor (for the child-adult interaction: drums, guitar, keyboard; for the adult-adult interaction: kangaroo, penguin, giraffe), black objects are unknown (for the child-adult interaction: saxophone, cymbals, maracas; for the adult-adult interaction: cassowary, axolotl, tarsier). Images below provide examples of interactions viewed from camera A when objects are present (situated context) and when they are absent (displaced context).

Finally, we asked speakers to talk about the objects when these were physically present (situated) or absent (displaced). This manipulation captures the observation that a large part of human communication is about objects and events that are absent from the current physical setting^53,54^. Indeed, displacement, i.e., language’s ability to refer to what is not here now, has long been considered as one of the design features of language^55^. However, we know very little about whether and how speakers modulate their speech and other multimodal cues based on displacement. Considering language acquisition, while there is growing attention to situating learning in caregiver-child interaction^56,57^ and therefore consideration of the multimodal behaviours by the caregiver and the child that can promote learning (e.g.^25,44,58–60^), only a few studies have explicitly investigated learning in displaced contexts^61–63^. In computational modelling, greater attention is currently placed on the development of multimodal models (or multimodal large language models, e.g.^64^) where multimodality refers to whether the model takes into account visual information from the context (in static and dynamic displays, e.g.^65^) in addition to language about those objects). Our corpus, contrasting the language (as well as the other multimodal signals) used when the visual context is situated and displaced can provide useful data to assess such models or fine-tune them in order to improve generalisability especially of child-oriented systems. Figure 1 provides a schematic of the corpus design.

## Methods

### Caregiver-child corpus

#### Participants

Forty-four caregiver-child dyads from the Greater London area (UK) participated in the study. Six dyads were excluded (camera malfunctioning, n = 1, child fussiness, n = 3, child had a hearing impairment, n = 1, failure to finish the experiment on the same day, n = 1). The mean age of children (N = 38; 19 girls and 19 boys) was 42.89 months, *SD = *4.54, range = 36-52 months. All remaining children who participated were typically developing, with no developmental delays reported by caregivers. Their mean score for a vocabulary test was 61.95 (range = 30-87, *SD = *13.44), as assessed using the British Picture Vocabulary Scale (BPVS3)^49^, administered right before the recording sessions. Two-thirds of children were the firstborn of the family (N = 25), and the rest were second (N = 11) and thirdborns (N = 2).

Caregivers (37 females, 1 male), being either the mother or father of a child, had a mean age of 38.45 years (*SD = *3.73), ranging from 29 to 48 years old. One caregiver had received a higher national certificate as their highest level of education, 19 had received a bachelor’s degree, 15 had received a master’s degree and 3 had received a doctorate. Caregivers’ professions included: teachers, sales/financial managers, journalists, CEO, researchers, and speech therapists (see details of the demographic information in the OSF project folder 10.17605/OSF.IO/H9CS6).

Caregivers reported that the family language (i.e., the language spoken by caregivers with the child in the home) was either British (N = 30) or American (N = 8) English. Caregivers were paid £30 for their participation and children were given a gift. Caregivers provided consent for data collection and sharing. Ethical approval was obtained from the University College London Research Ethics Committee (ECOLANG 143/002).

#### Materials

Objects (toys) provided to dyads were from 4 categories: foods, musical instruments, animals, and tools. These categories are common for children of this age range and were chosen for the availability of toys and for the opportunities these categories offer for vocal and manual iconicity. We developed a pool of 98 toys across the four categories. The word frequency of object labels^66^ was normally distributed *D* = 0.057, *p* = 0.89 (*M* = 3.71, *SD* = 0.76, range = 1.54–5.17). The mean word frequency for unfamiliar words (*M* = 3.30, *SD* = 0.69, range = 1.54–4.76) was significantly lower than for familiar words (*M* = 3.87, *SD* = 0.68, range = 1.74–5.17), *t* (137) = 4.82, *p* < 0.001). To increase the generalizability of our study, we included all our toys without restricting words to a specific stress pattern of the same number of syllables. The mean number of syllables of the unfamiliar words for toys in the interactions was 2.14 (*SD* = 0.97, range = 1–4) and the mean number of syllables of familiar ones was 1.93 (*SD* = 0.91, range = 1–4). For each dyad, we used 6 toys from each category, with 3 toys familiar (both concept and label) to the child, and 3 unfamiliar (both concept and label), based on parental reports of the child’s knowledge. On occasion, there were instances where an object was familiar to the child, but the caregiver reported it as ‘unfamiliar’. Based on video recordings, there were ten clear instances where children were already familiar with an object before the interaction, which have been noted. Individual toys were used in a roughly equal number of testing sessions across participants. A full list of the toys, the toys used for each dyad with their familiarity to children, as well as the familiarity notes can be found in the folder ‘objects used in the study’ of the OSF project.

#### Procedure

Recording sessions took place in the families’ homes. Caregivers were contacted several days before the session with a full list of toy names and asked to indicate whether their child knew each object and each word without checking with their child. The assignment of toys to the familiarity conditions was based on these responses. Two experimenters then visited the family and recorded the interactions using: two video cameras (Panasonic V250), one of which focused on the caregiver, the other of which focused on the interaction space capturing both the child and the caregiver, a unidirectional lavalier (lapel) microphone and eye tracker glasses (Tobii Pro Glasses 2) for the caregiver. Note that lapel microphone recordings are not included in the available corpus because they are not anonymised. The audio of the session is available from the video camera alone, in which names of people have been muted. A clapperboard was used for synchronisation of the different devices.

During recording, one experimenter monitored the correct working of the equipment, but left the room. The second experimenter brought and removed the toy sets to and from the interaction space. The interactions were carried out at a table, with the caregiver and child sitting at 90 degrees from each other (see Fig. 1). Caregivers were asked to interact with their child in a natural way, but to try to talk about each of the objects provided. A list with the toys’ names was given to the caregivers at the beginning of each category round to help them remember the toys in the set during the toy absent manipulation. The order of the object present/absent manipulation was counterbalanced across participants.

When the object present manipulation came first, the experimenter brought a set of 6 toys from one category (e.g., animals) to the table and left the room. The caregiver and child talked about these objects (3-5 minutes), before the experimenter returned and removed the toys from the interaction space, by asking the child to “*help them tidy up so that she can bring a new set of toys*”. The experimenter left the room with the toys for the object absent manipulation, asking the caregiver to continue talking about the toys they had just seen (3-5 minutes). Specifically, they said: “*while I go and look for a new set of toys, why don’t you continue to chat about those you just had?*”. The experimenter then brought in a new set of 6 toys, repeating this procedure for all 4 toy categories.

When the object absent manipulation came first, the experimenter said: “*I am going to get some toys for you, while you wait, why don’t you start talking about the toys that I am about to bring?*”. After 3-5 minutes, the experimenter brought in the set of toys for that category, and left the room, leaving the caregiver and child to interact with the objects present. After 3–5 minutes, the experimenter entered the room to remove the toys and the procedure continued for all 4 toy categories. The full recording session lasted approximately 35–45 minutes.

The order of presentation was counterbalanced for two main reasons. First, if the object-absent condition always follows the object-present, all representational gestures in the corpus could be the result of priming from object manipulations in the object-present condition, rather than more strongly engage representational processes in the speaker (and in the addressee). Second, counterbalancing allows us to better tap into how speakers introduce referents unknown to their addressee (child or adult) in situated and displaced contexts. Unknown objects are truly unknown only during the first session (being displaced or situated) thus we wanted to be able to capture differences in speakers’ behaviours related to displacement during this session.

### Data processing

Audio-visual data was annotated for caregiver communicative behaviours according to the annotation protocol described below (see the ECOLANG manual https://osf.io/4rv7n for more details). Audio (speech), video (gestures) and eye-tracking (gazing) data streams were annotated separately, and then combined. To make the children unidentifiable and follow ethics regulation, all children’s faces were manually blurred using the software Adobe After Effect^67^ or DaVinci Resolve^68^, and any names related to their identities were muted.

### Speech annotation

Caregiver speech was initially manually transcribed and segmented by utterance. An utterance was defined as a unit that expresses a single situation (activity, event or state) with an implicit or explicit predicate^69^. Examples of utterances are: “I eat the bread”; “Red” (meaning “It is red”); “Push” (see more in the ECOLANG manual p. 6).

For each utterance, we annotated the topic, namely the specific toy (or multiple toys) that the utterance referred to. For example, the topic would be noted as “saxophone”, if the caregiver said “Can you play the saxophone?” or “Can you play it?” when referring to the saxophone, or if they pointed to the saxophone and said “What’s that?”. In cases where there was more than one topic (if the caregiver was communicating about more than one toy, or the topics communicated by speech and manual cues differed), the different topics were coded. The topic coding provided our basis for classifying utterances as familiar or unfamiliar. Utterances that did not focus on our toy referents were coded as “other”. We also annotated whether the explicit label was produced (i.e., when the caregiver produced the name of the toy). Utterance-level coding was carried out by trained members of the research team using Praat^70^. Explicit mentions of toys’ labels (i.e., the caregiver saying the word “saxophone”) were extracted from the transcriptions and annotated to be temporally aligned with the utterance in a separate tier of the Praat textgrid. Any names related to the participants were not transcribed but were replaced with “<name>”. Additionally, laughter was coded using “<laugh>” and sound effects using “<noise>”. Unclear speech (where coders could not discern what was being said) was marked as “<unclear>”.

We separately annotated onomatopoeia, which included both lexicalised onomatopoeia (e.g., in the phrase “The dog goes *woof*”) as well as sound effects produced by the mouth (e.g., making a barking noise). Detailed instructions for coding onomatopoeia can be found in the ECOLANG manual (p.9). Onomatopoeia coding was carried out by trained members of the research team.

Fillers and interjections were transcribed according to conventions (detailed in the ECOLANG manual, p.15). Note that utterances in the ECOLANG dataset do not begin with filler or interjections, that is to say that utterances begin at the first contentful word that expresses the single event. As such, the speech “um this is a chatelaine” would be two utterances (“um” and “this is a chatelaine”). However, if filler words or interjections occur mid-event (“this is a um chatelaine”) they are not segmented into two utterances. After transcription, all utterances containing filler or interjections were located using this controlled vocabulary and marked on separate tiers. Additionally, utterances that consisted solely of filler or interjections were also identified and marked (e.g., utterances containing simply “um”). This is to allow researchers using the corpus to filter these annotations out.

### Manual cues’ Annotation

Data from all caregivers were coded for the following manual cues.

1. Representational gestures
  Iconic and metaphorical gestures that imagistically represent properties of referents. This may be through depicting the shape or size of an object (e.g., hands moving apart to represent the long legs of the ostrich), how the object is manipulated (e.g., a hammering gesture in which the shape of the hand represents how a hand holds a hammer), or gestures that metaphorically represent an abstract concept (e.g., hand moving away from the body to represent something far away). Enumerative gestures (e.g., holding up fingers to count objects) are included in representational gestures, however have been marked on a separate tier to allow researchers to filter these.
2. Points
  Gestures that single out a referent through deixis. These may be a canonical index finger point, or an open palm gesture indicating a specific object. The majority of points are directed at particular objects and occur in the toy present condition. As such they allow the communicator to create a visual link to an object. Points can also appear in the toy absent condition (e.g., to a location the object previously occupied, or to an object that acts as a stand-in for another object), though much less often. Such points cannot establish the same visual link, so they may have a different representational characterisation and function.
  Occasionally, speakers would point to the list of objects that they were talking about when referring to an object named on the cue list. To allow researchers to filter these instances out, we have identified all points to the list.
3. Beat gestures
  Repeated up-down movement of the index finger/hand^71^. Beat gestures are often aligned with the rhythm of the speech that stresses what is being talked about)^72^. Note that beat gestures were only coded when they were produced alone. Of course, people might produce beat gestures while producing other gestures, (e.g., representational gestures, if someone emphasises an element of the gesture) but these were not coded as beat gestures. Beat gestures were annotated when there was no clear referential or pragmatic function.
4. Pragmatic gestures
  These are also called ‘interaction gestures’. They do not convey semantic content, but manage and express the communicative interaction between listener and speaker^73^ (see the ECOLANG manual p.22 for more details).
5. Object manipulations
  Object manipulations are defined as actions or movements performed while holding or manipulating an object. For example, this could be the caregiver holding an object with the intention of making it visually salient to the child, or performing an action on an object, such as tapping the keys on the keyboard to illustrate how the object is used/functions. We only coded instances in which the speaker is holding the object or manipulating it for the purpose of communication, and excluded instances where speakers manipulated objects in non-communicative ways, for example to pick it up from the floor, to move it aside so to be able to reach another object, or simply they are fidgeting with it (perhaps absent-mindedly).
  Object manipulations were only coded in the toy present condition and only for the toys we provided. A subset of participants (N = 21) had an initial pass through the crowd-sourced coding, using the Gorilla experiment builder^74^, via Prolific (www.prolific.co). Online coders were shown a few introductory video clips of parent-child interactions and were given instructions about how to code them. Each online coder saw a total of approximately 150 video clips, each lasting less than 30 seconds. For each video clip, the online coder was asked: (1) to classify the caregiver’s hand gesture into either representational gesture, object manipulation, point, or other (if it did not fall into any of these categories), and (2) to identify the object that the gesture referred to (to select one of the available objects, or none if they could not identify the object). The procedure took about 30 minutes and participants were paid £3.75 for participation. Each video clip was seen and coded by at least 7 online coders. The answer of the majority was taken as the selected answer (i.e. more than 60% of online coders had to agree on the gesture or object identity). As the agreement between online coders was not high, the coding of these 21 participants and the rest of the corpus (N = 18) was finally coded by expert coders following the coding scheme above.
  In addition, all gestures were assigned a topic. For representational gestures, pointing and object manipulations, the topic was coded according to the referent. For pragmatic/beat gestures the topic was coded according to the accompanying speech (as they do not obviously have content).

### Eye gaze

Eye gaze data were collected for all caregivers except Ch08 due to a technical error. Eye gaze raw recordings were first processed using Python to mark gaze position in the caregiver point-of-view recording obtained from the eye tracking glasses. Then the video was annotated by an expert coder manually, noting the specific toy that was the focus of gaze fixation. The gaze fixation was coded for gazes that lasted for 3 or more consecutive video frames. As gaze was coded for fixation on specific objects, it was only coded for the toy present condition.

### Multimodal integration

As mentioned above, audio, video and eye-tracking data were coded separately in Praat (speech), and in ELAN (download link https://archive.mpi.nl/tla/elan)^75^ (manual gestures and eye-gaze), and then combined in ELAN. To do this, first the multimedia files were aligned — the signal for the beginning of recording was a clapperboard snap which was easily detectable in video and audio channels — and then the codings were offset based on the start point of the recording and imported to ELAN. Note that for researchers interested in working in Praat (for example if they wanted to take advantage of Praat’s tools for speech analysis), the data can be exported from ELAN in the form of a Praat textgrid (File > Export as > Praat TextGrid), which would synchronise with audio extracted from the video.

### Adult-Adult corpus

#### Participants

34 dyads of native speakers of British (N = 29) or American (N = 5) English participated in the study. Each dyad had a speaker and an addressee, who knew each other before the experiment. Data from one dyad were excluded as they did not understand the task. For the remaining 33 dyads, the mean age of speakers (19 females and 14 males) was 24.24 years, range 18-43, *SD* = 6.15, and of addressees (17 females and 16 males) was 24.76 years, range 18–47, *SD* = 6.29. The majority of them had higher education (speakers: High school = 2 (6.06%), Bachelor = 20 (60.60%), Master = 8 (24.24%), Doctorate = 3 (9.09%); addressees: High school = 3 (9.09%), Bachelor = 19 (57.58%), Master = 8 (24.24%), Doctorate = 3 (9.09%). Participants received payment (speakers: £20; addressees: £10) for taking part in the study. Participants provided consent for data collection and sharing except two speakers disagreed to publish the recordings. Thus, we finally included 31 dyads in the corpus.

#### Materials

The objects were drawn from the same 4 categories as in the caregiver-child corpus (foods, musical instruments, animals, and tools). In total, there were 37 objects, including 19 that were generally unfamiliar and 18 that were generally familiar. For each dyad, 24 objects were selected, with an equal split between familiar and unfamiliar objects. These were evenly distributed across the categories, resulting in 3 familiar and 3 unfamiliar objects per category.

The selection of familiar and unfamiliar objects followed a two-step process. First, we selected 8 familiar objects used in the caregiver-child corpus. Second, we generated a list of 81 familiar and unfamiliar objects and conducted an online norming survey with 51 native English speakers in the UK and US. For each object, participants were asked to rate on a 5-point scale about: (1) the extent to which they are familiar with the label (object name (label)); (2) the extent to which they are familiar with the object (presented as a picture); and (3) indicate whether they find the object interesting (Y/N). This latter measure was introduced identify objects that participants may find more engaging to discuss (those that are more generally interesting). The final list included unfamiliar objects with a mean familiarity score of 2.35 (*SD* = 0.93) for picture ratings and 1.47 (*SD* = 0.39) for label ratings, as well as familiar objects with a mean familiarity score of 4.68 (*SD* = 0.54) for picture ratings. On average, 61% (*SD* = 15%) of participants indicated that familiar objects were interesting, while 65% (*SD* = 18%) reported the same for unfamiliar objects. Detailed rating scores for each object can be found in the OSF folder ‘objects used in the study’.

Finally, as the number of unfamiliar objects in the musical instrument category was fewer than other categories, we added two unfamiliar objects (caxixi and shekere). Their familiarity was only informally assessed among several English colleagues (note that familiarity status was further confirmed in a post-experiment survey of English addressees).

For each unfamiliar object, we created a training video introducing the object (where it comes from, what/how it is used and other information about it) and its name. The average length of the video was 1.5 min, range 1-2. We also prepared a digital picture of the unfamiliar objects, accompanied by their orthography, International Phonetic Alphabet (IPA) and pre-recorded label pronunciation by a native speaker of British English. For each familiar object, we prepared a slide with a photo, a summary (as a series of bullet points) of general information about it, and its name. These training materials were set up in a Qualtrics survey so that participants could watch them at home before coming to the lab for the interaction session. The full list of objects used for each dyad, pictures of the unfamiliar objects, and video training materials can be found on the project’s folder ‘objects used in the study/adult-adult’.

#### Procedure

One or two days before the experiment, the ‘speaker’ (i.e., the participant signing up for the experiment) was sent the Qualtrics survey with the descriptions of the objects to be used in the interactions (objects were randomly assigned to each speaker). The speaker was asked to study these materials carefully so that they could talk about the objects with their ‘addressee’ (i.e., the person who was invited to the experiment by the speaker) in the study. However, the speaker was instructed not to tell the addressee about the objects before the interaction session.

On the testing day, both the speaker and addressee visited a lab room in the Experimental Psychology Department at UCL. Right before the recording session, the speaker was asked to go over a PowerPoint presentation listing all the objects for the experiment (including the pronunciation of their names). The interaction session was recorded with two video cameras (Panasonic V250), one focused on the speaker, the other on the interaction space. Both the speaker’s and the addressee’s speech were recorded with a unidirectional lavalier (lapel) microphone (note this is not included in the corpus but the audio is available from the video camera alone). The speaker’s gaze fixations were recorded with Tobii Pro Glasses 2 and the addressee’s gaze fixations were recorded with a Pupil labs eye tracker. A clapperboard was used for synchronisation of the different devices.

During recording, one experimenter monitored the correct working of the equipment from an observation room where participants could be seen through the one-way transparent glass. The second experimenter brought and removed object sets to and from the interaction space. The interactions were carried out at a table, with the speaker and addressee sitting at 90 degrees from each other, talking about each of the objects provided.

A typed list of the objects was given to the speaker at the beginning of each category to remind them of all the objects in the set during the object absent manipulation. The procedure of presenting objects of four categories was the same as that of the caregiver-child interaction (see procedure in caregiver-child corpus). The full recording session lasted approximately 35–45 minutes.

### Additional testing for addressee and speaker

Vocabulary Measure (addressee and speaker).

An English vocabulary test was carried out with both the speaker and addressee. We chose the Ghent University Vocabulary test because of its length (The introduction of the test can be found here http://crr.ugent.be/archives/1533. The original link to the online test was http://vocabulary.ugent.be/wordtest/start but it is no longer available): Each participant saw 100 letter sequences, some of which are existing English words and some of which are made-up nonwords. Participants indicated for each letter sequence whether it is a word they know or not. This was carried out on a laptop provided by the experimenter. Results were calculated as a percentage score. The mean score for speakers was 64.03%, *SD* = 0.110, range 42%-84%; the mean score for addressees was 65.35%, *SD* = 0.124, range 36%-83%. Participants’ background information such as age, education, and how many languages they speak was also collected in this task (see ‘ECOLANG demographics’ in the OSF project folder).

### Post-study survey

At the end of testing, both speakers and addressees were given a list of unfamiliar words that were used as experimental stimuli in the interaction. Speakers were asked to indicate whether they thought that their addressee knew the unfamiliar words (label and concept) before the session. Addressees were asked to indicate whether they knew any of the unfamiliar words before the interaction. The results showed that on average speakers thought that addressees knew 1.74 unknown objects (*SD* = 1.37), which was correlated with addressees’ report (*M* = 1.65, *SD* = 1.23), *r (29)* = 0.70, *p* < 0.001. Additionally, speakers believed that addressees knew some concept (but not the label) of 0.19 unknown objects (*SD* = 0.60), correlating with addressees’ report as well (*M* = 0.29, *SD* = 0.74), *r (29)* = 0.54, *p* < 0.002. The details of this information for each dyad can be found in the OSF project folder ‘Objects used in the study’.

### Data processing

#### Speech coding

The speaker’s speech was initially automatically transcribed using temi/google speech-to-text. The transcription was then manually checked, corrected, segmented, and aligned with the speech at the utterance level in Praat.

Following the caregiver-child corpus, we coded the four object categories and displacement (present/absent) for each recording, giving 8 sections per transcription. We coded the topic for the specific object(s) that each utterance referred to. Additionally, we automatically extracted explicit mentions of the label of each stimuli object in a separate tier, which was also manually checked and corrected. All speech annotations were done in Praat.

#### Manual cues

We applied the same coding criteria used for caregivers to code manual cues of gestures and object manipulations from speakers in ELAN. Eye gaze data were collected for both speakers and addressees, but only data of speakers were processed. Two speakers were excluded due to technical malfunctions. After these exclusions there were gaze data from 29 speakers in the corpus. The multimedia files were aligned as the child-directed corpus (see section Multimodal integration).

## Data Records

**Fig. 2:**
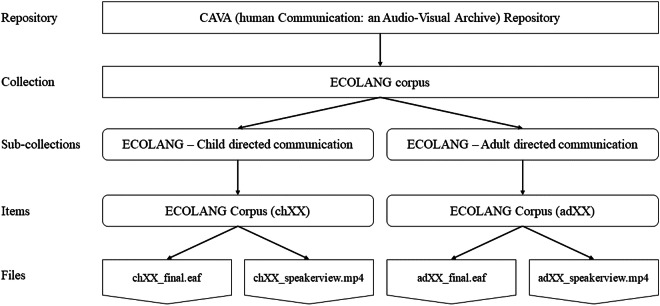
Visualisation of the data structure.

The ECOLANG corpus can be accessed at the CAVA (Human Communication: An Audio-Visual Archive) repository^76^ at UCL 10.5522/04/28087613, with additional resources (e.g., the coding manual) on the OSF (10.17605/OSF.IO/H9CS6) associated with the corpus^77^. To protect the participants’ privacy, users will need to sign a user agreement to have full access to the data. The structure of the data collection is illustrated in Fig. 2. At the top level, there is the ECOLANG corpus collection, which comprises two sub-collections: child-directed communication and adult-directed communication. The sub-collection related to child-directed communication consists of 38 item folders, each labelled as ‘chxx’ (e.g., ch07). Each of these folders contains data about a caregiver-child dyad, including the final multimodal integrated annotation Elan file (e.g., ch07_final.eaf) and the corresponding matched video (e.g., ch07_speakerview.mp4). Note that the child’s face in the videos is blurred in compliance with the ethical requirements of the funder. The corpus does not contain the second video from the interaction view because children’s faces in these videos have not been blurred, and children (with their faces blurred) are mostly visible in the ‘speakerview’ videos.

The subdirectory related to adult-directed communication contains 31 item folders labelled as ‘adxx’ (e.g., ad01). Each of these folders contains data related to adult-adult dyadic interactions. Within each item folder, there are the final multimodal integrated annotation Elan files (e.g., ad01_final.eaf) along with their corresponding video (e.g., ad01_speakerview.mp4). Similar to the child-directed sub-collection, these videos capture interactions where the speaker is always visible and the addressee is mostly visible.

## Technical Validation

Table 1 provides information on the duration of the sessions from which annotations are derived while Table 2 presents the number of annotations (mean, median, and range) for all coded behaviours, including spoken utterances, gestures, and gazes directed towards objects. These data are about information found within the ‘Topic’ tier, gesture topic tiers (e.g., ‘Topic_RepGest’), ‘Object’ tier (indicating gazes towards objects), as well as instances where behaviours referred to entire sets of objects, and those marked as ‘other’ that signifies behaviours unrelated to or not directed towards any of the objects.

**Table 1:** Length of Sessions (s) across the ECOLANG corpus.

Objects	Presence	Child-directed		Adult-directed	
		*M* length (Med) (s)	Range (s)	*M* length (Med) (s)	Range (s)
Animals	Present	272.73 (266.27)	224.99–346.12	294.25 (276.80)	243.02–611.47
Animals	Absent	247.67 (247.95)	174.82–387.46	269.14 (274.14)	198.66–315.65
Food	Present	267.28 (266.98)	215.74–368.42	286.31 (276.57)	226.54–447.52
Food	Absent	237.66 (229.71)	183.78–354.28	262.75 (259.81)	190.08–305.51
Music	Present	286.09 (280.59)	238.88–373.25	305.87 (290.72)	242.97–568.55
Music	Absent	241.03 (238.24)	154.92–358.94	263.62 (262.04)	128.90–409.54
Tools	Present	280.18 (270.38)	182.22–411.92	303.76 (284.08)	245.30–459.08
Tools	Absent	233.89 (238.31)	120.72–280.29	269.66 (264.47)	195.03–327.86

**Table 2:** Frequency of annotations in the ECOLANG corpus for behaviours.

Behaviour	Tier name in ELAN file	Child-directed		Adult-directed	
		*M* (Med)	Range	*M* (Med)	Range
Utterances	Speaker	789.92 (802.5)	542–1120	854.39 (850)	619–1426
Containing Labels	SpeakerReferent	165.18 (156)	87–258	94.03 (90)	50–144
Containing Onomatopoeia(s)	Onomatopoeia	25.13 (24)	6–67	n/a	n/a
Containing Interjection(s)	interjection	75.21 (69.5)	26–166	41.71 (37)	16–95
Interjection only	interjection_only	71.05 (67.5)	21–166	37.58 (35)	14–80
Containing filler(s)	Filler	26 (22)	2–85	139.19 (130)	45–271
Filler only	filler_only	24.58 (21.5)	2–82	108.97 (101)	32–196
Representational Gestures	RepGest	44.26 (40.5)	16–134	112.42 (97)	32–258
Enumerations	RepGest_enumeration	5.29 (2.5)	0–44	1.97 (1)	0–10
Pointing	Point	27.92 (26)	3–81	44.42 (42)	10–105
Pointing to list	Point_list	0.92 (0)	0–8	2.03 (1)	0–12
Object Manipulations	ObjMan	80.16 (80)	36–142	71.97 (67)	35–183
Pragmatic Gestures	PragGest	14.16 (10)	2–69	94.68 (85)	12–183
Beat Gestures	BeatGest	3.87 (3)	0–20	19.58 (11)	1–99
Gaze to Objects	Object	761.5 (716)	0–1825	554 (599)	0–1273

Note. Onomatopoeias are not coded in adult-directed speech. For every annotation on the “Speaker” tier, there is a corresponding annotation on the “Topic” tier, and for annotation on a tier relating to a gesture (e.g., “RepGest”) there is a corresponding annotation on the tier relating to that gesture’s topic (e.g., “Topic_RepGest”).

### Reliability of annotations

To ensure the reliability of subjective coding for behaviours such as **gestures,** **object manipulation,** **gaze** towards objects, and **onomatopoeia**, inter-rater reliability assessments were conducted.

For gestures, and object manipulations a second trained coder, blind to the original coding, annotated these cues in a randomly selected portion of the video, which represented approximately 10% of the entire interaction (across both corpora, this resulted in approximately 4 hours and 25 minutes of double-coded video).

For Representational Gestures, Pointing, Pragmatic and Beat Gestures (where count data are the most informative), agreement between the coders on the number of gestures produced was high (adult-child corpus, r = 0.815; adult-adult corpus, r = 0.850). In addition, the timing of gestures was reliable, when comparing gesture coding within 500 ms bins across the coders (gesture coded within that 500 ms = 1, not = 0; adult-child corpus, 94.49% agreement, κ = 0.764 [95% CI = 0.749–0.779]; adult-adult corpus, 92.32% agreement, κ = 0.764 [95% CI = 0.749–0.779]). For agreed gestures (where the reliability coder coded a single gesture overlapping in time with the original coding), there was good agreement on the type of gestures: adult-child corpus, 93.05% agreement, κ = 0.885 [95% CI = 0.835–0.935] across n = 273 gestures (a further n = 38 gestures were not considered as the reliability coder did not code an overlapping gesture, and n = 29 were not considered as they involved multiple overlapping gestures); adult-adult corpus, 82.53% agreement, κ = 0.717 [95% CI = 0.674–0.760] across n = 734 gestures (a further n = 78 gestures were not considered as the reliability coder did not code an overlapping gesture, and n = 142 were not considered as they involved multiple overlapping gestures). It is worth noting that in the adult-adult corpus, the majority of disagreements arose over distinguishing pragmatic gestures both from beat gestures and from representational gestures. Many researchers opt to collapse beat gestures with pragmatic gestures (following^73^) whereas here we attempted to retain functional differences between the gestures. Collapsing these categories improves reliability slightly (85.83% agreement, κ = 0.766 [95% CI = 0.724–0.808]). Furthermore, we believe that these disagreements reflect the truth that distinguishing some representational gestures (those potentially involving metaphor) from pragmatic gestures is somewhat subjective. In the adult-child corpus, this was not such an issue, as pragmatic and beat gestures were rare (see also^78,79^), and we speculate that more complex representational gestures (i.e., conduit metaphors) are less common.

For Object Manipulations (where data on duration is most informative), there was also a very high agreement between the two coders on the duration of object manipulations (adult-child corpus, *r* = 0.927; adult-adult corpus, *r* = 0.974). In addition, the timing of object manipulations was reliable, when comparing coding within 500 ms bins across the coders (adult-child corpus, 93.71% agreement, κ = 0.748 [95% CI = 0.733–0.763]; adult-adult corpus, 92.28% agreement, κ = 0.802 [95% CI = 0.791–0.812]).

Coding for the speaker/caregiver’s gaze towards objects was conducted by the second coder for roughly half of the dyads (19 adult-child dyads and 17 adult-adult dyads) for 120 s per dyad in a randomly selected session (out of the 4, beginning coding 60 s after the session started). Agreement between the coders on the number of fixations on objects was high (adult-child corpus, *r* = 0.940; adult-adult corpus, *r* = 0.781). The timing of gaze was reliable when comparing gaze coding within 500 ms bins across coders (gaze to object coded within that 500 ms = 1, not = 0; adult-child corpus, 91.62% agreement, κ = 0.832 [95% CI = 0.816–0.849]; adult-adult corpus, 86.93% agreement, κ = 0.728 [95% CI = 0.706–0.750]). For agreed fixations (where both coders coded a fixation overlapping in time with the other coder), there was excellent agreement on the target of the fixation (adult-child corpus, 96.33% agreement, *κ* = 0.962 [95% CI = 0.950–0.973] across *n* = 1090 fixations; adult-adult corpus, 90.17% agreement, *κ* = 0.895 [95% CI = 0.869–0.921] across *n* = 590 fixations). It is worth noting that discrepancy in agreement across the corpora may arise simply because the objects were on the whole smaller for the adult-adult dyads compared to the adult-child dyads, making determining the target of the fixation harder.

Onomatopoeia reliability coding was conducted for 19 adult-child dyads on a randomly selected decile of utterances, with the second coder marking all sound effects and lexicalised onomatopoeia. Agreement between the coders on the number of onomatopoeias produced was high (*r* = 0.957).

### Effect of manipulations

To demonstrate that the manipulations in the ECOLANG corpus (audience, object familiarity and presence) have an impact on annotated behaviours, we present the variation in a number of behaviours across the manipulations in the experiment. We focus on contentful utterances (i.e., excluding filler only and interjection only utterances) about single objects (i.e., excluding utterances about multiple objects and utterances, not about the objects [marked “other” on the “Topic” tier]).

**Fig. 3:**
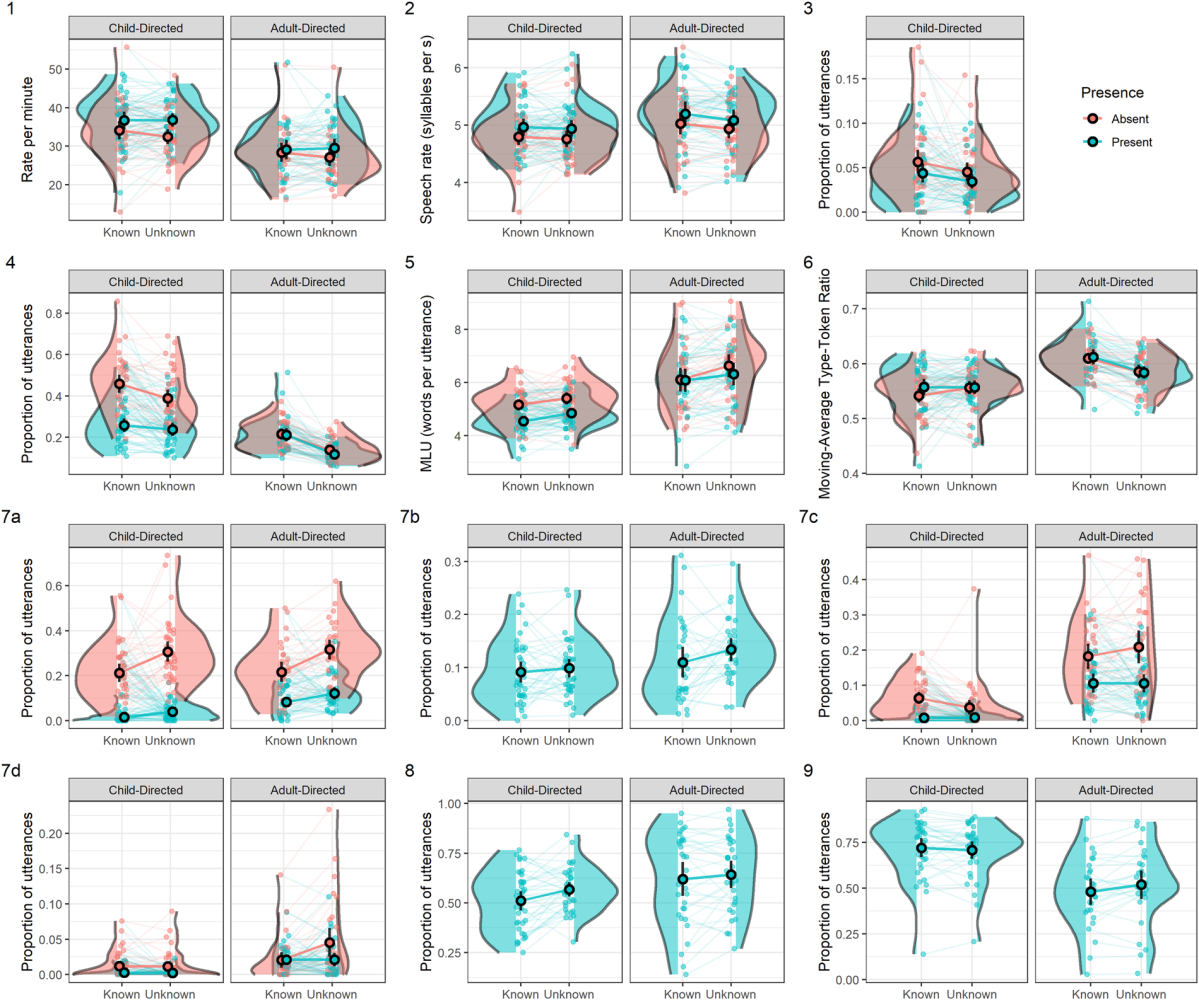
Variation in multimodal behaviours across the manipulations in the experiment. (1) Rate of utterances per minute, (2) speech rate, proportion of utterances containing (3) onomatopoeia (CDL only) or (4) containing object labels; (5) mean length of utterances (words per utterance); (6) lexical diversity (MATTR) of speech; proportion of utterances co-occurring with (7a) a representational gesture, (7b) a pointing gesture (present condition only), (7c) a pragmatic gesture, (7 d) a beat gesture, (8) an object manipulation (present condition only), and (9) a gaze to an object (present condition only). Overlaid point ranges denote mean and confidence intervals across all participants, with coloured lines linking these between object familiarity.

The annotated behaviours include: (1) rate of utterance per minute (number of utterances produced in a minute of speech about an object). To account for different session lengths (and the amount of time participants may choose to spend discussing the objects), we considered continuous speech about an object as occurring when consecutive utterances were on the same topic and not separated by more than 1 s, with the onset being the onset of the first communicative behaviour in the block and the offset being the offset of the last communicative behaviour in the block (total duration of all utterances of the same topics plus pauses less than 1 s), (2) speech rate (number of syllables in utterance/utterance duration (s)) where the number of syllables per utterance is estimated using nsyllable in R^80^), (3) proportion of utterances that contain onomatopoeia or (4) object labels, and (5) mean length of utterances (length in words, as in^81,82^, see also^83^), (6) lexical diversity (the moving-average type-token ratio [MATTR] using a window of 100 utterances, calculated using koRpus::MATTR^84^) and the proportion of utterances overlapped in time with (7a) representational gestures (excluding enumerations), (7b) points (excluding points to the list), (7c) pragmatic gestures, (7 d) beat gestures, (8) object manipulations, and (9) gazes to objects (see the visualisation in Fig. 3). For more information about the details for each category of the multimodal behaviours (see Supplementary Information).

## Usage Notes

To request access to the ECOLANG data, researchers need to apply for a user licence by contacting the Project Officer (ecolang@ucl.ac.uk). The CAVA team (cava.admin@ucl.ac.uk) will issue a login that will give access to all the publicly available data once the user has signed the user license agreement. The user licenses are issued in batches every few weeks and remain active until the end of the calendar year. If a user license has expired (for example, if a login from a previous year is no longer valid), one can reapply (see more details at the CAVA FAQs https://www.ucl.ac.uk/ls/cava/faq.shtml and ECOLANG on CAVA https://www.ucl.ac.uk/pals/ecolang-corpus/ecolang-cava).

On inspection of the data, some speakers have few gazes co-occurring with speech, which results from eye-tracking malfunction (either poor calibration or a fit too high on the participants’ head, meaning the table with objects was not often in view for the eyetracker scene camera). We would recommend excluding participants from the data who have < *M* – *SD* fixations coded from analyses of gaze data (ad04, ad08, ad14, ad32 and ch34).

Since the familiarity of unknown words was determined based on the caregiver’s report, there are slight differences compared to the actual familiarity status perceived by the children. Users may want to consider using familiarity statuses that have been adjusted based on the video interaction to more accurately reflect the children’s perception. Similarly, for unfamiliar objects in adult-adult interactions, users can refine the familiarity status of these objects based on the results of post-experiment surveys.

The goal of the corpus is to capture the beahviours by the speaker and therefore the video recordings are optimized for this purpose. There are some periods where the child’s hands or face are out of view, which somewhat reduce the opportunity to carry out additional annotation of the child’s manual behaviours. If users wish to study details of children’s multimodal behaviours, they may make additional annotations as to the availability of the modalities. They can also get in touch with us so that we could discuss collaborative arrangements in order to be able to share non-anonymized videos of the interaction (“both view”) where children’s face and body behaviours are clearly visible.

## Supplementary information

Supplementary Information

## Data Availability

The written code (R scripts) to pre-process the effect of manipulation data is included in the project OSF folder ‘code’.
