# Supplementary material for: The ECOLANG Multimodal Corpus of adult-child and adult-adult Language
Source: Sci Data. 2025 Jan 16;12:89. doi: 10.1038/s41597-025-04405-1 (PMC11739475; doi:10.1038/s41597-025-04405-1)
Supplement: Supplementary file 1 — Supplementary Information [file 41597_2025_4405_MOESM1_ESM.docx]

**Supplementary Information**

Figure 1a. Variation in (1) utterance rate, (2) speech rate, and (3) proportion of onomatopoeia (Child-directed corpus only) across the manipulations in the experiment split by category.


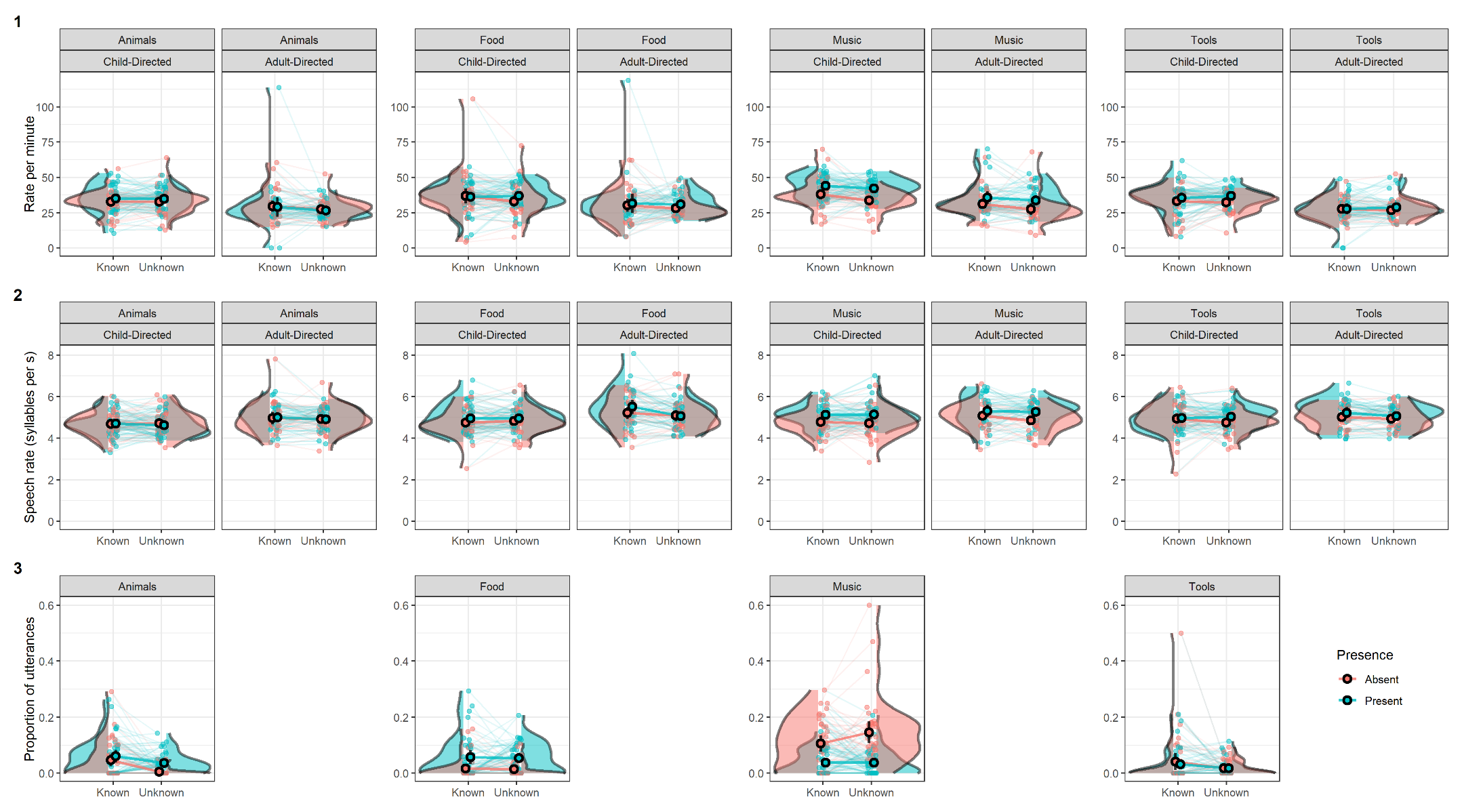


Figure 1b. Variation in (4) proportion of object label (5) mean length of utterance (MLU), and (6) lexical diversity across the manipulations in the experiment split by category.


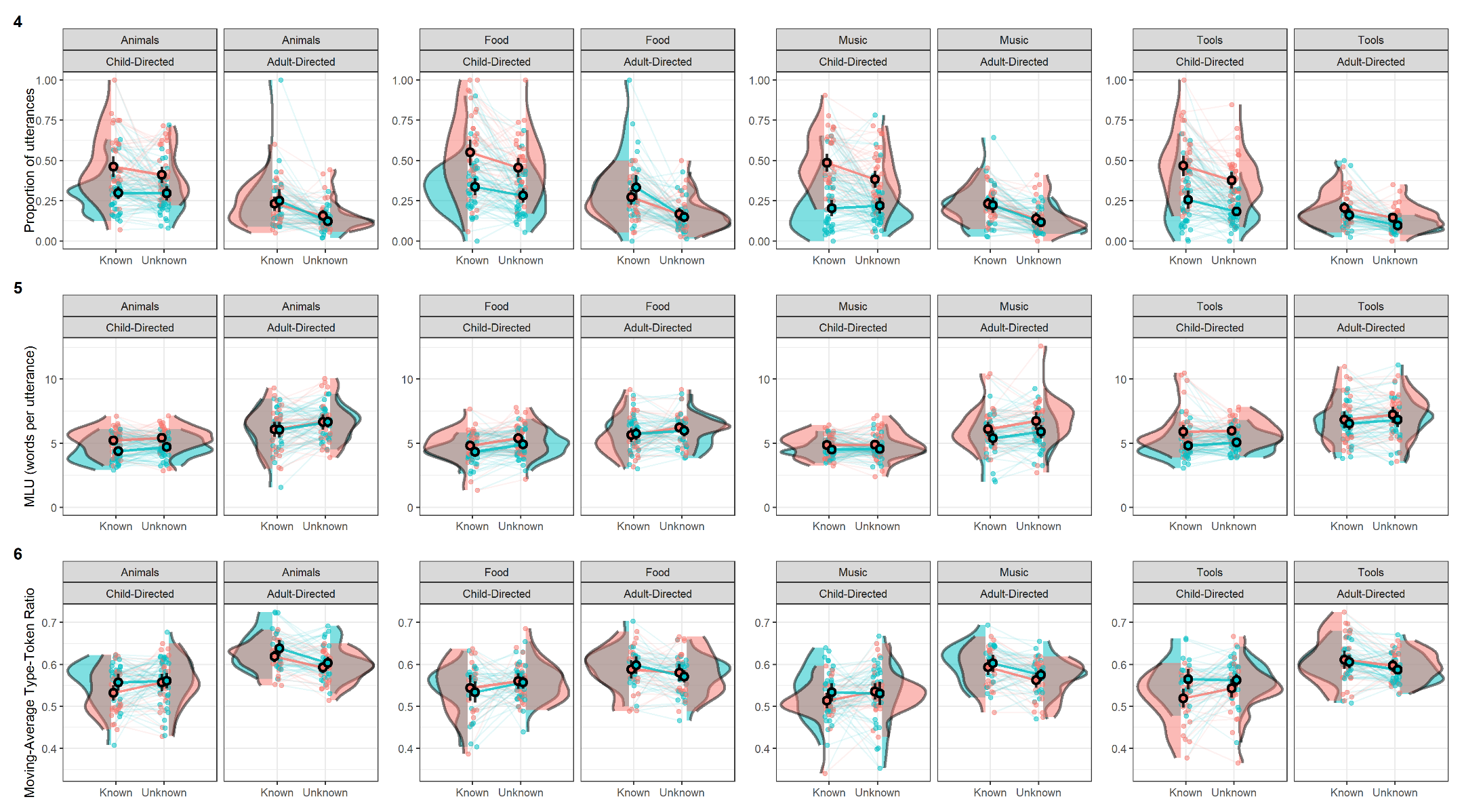


Figure 1c. Variation in the proportion of utterances co-occurring with a (7a) representational gesture (7b) pointing gesture (present condition only), and (7c) pragmatic gesture across the manipulations in the experiment split by category.


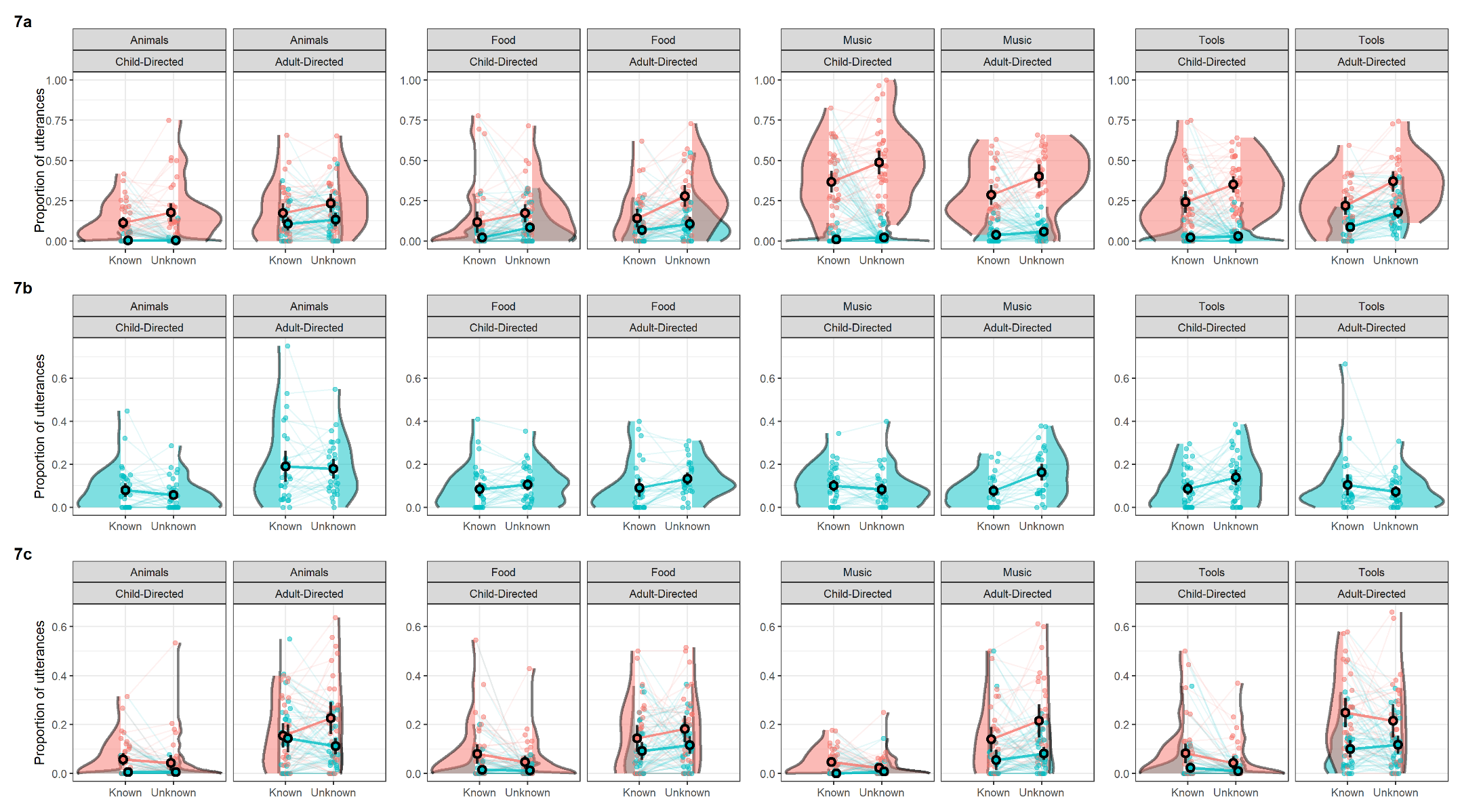


Figure 1d. Variation in the proportion of utterances co-occurring with (7d) a beat gesture (8) an object manipulation (present condition only), and (9) a gaze to an object across the manipulations in the experiment split by category.


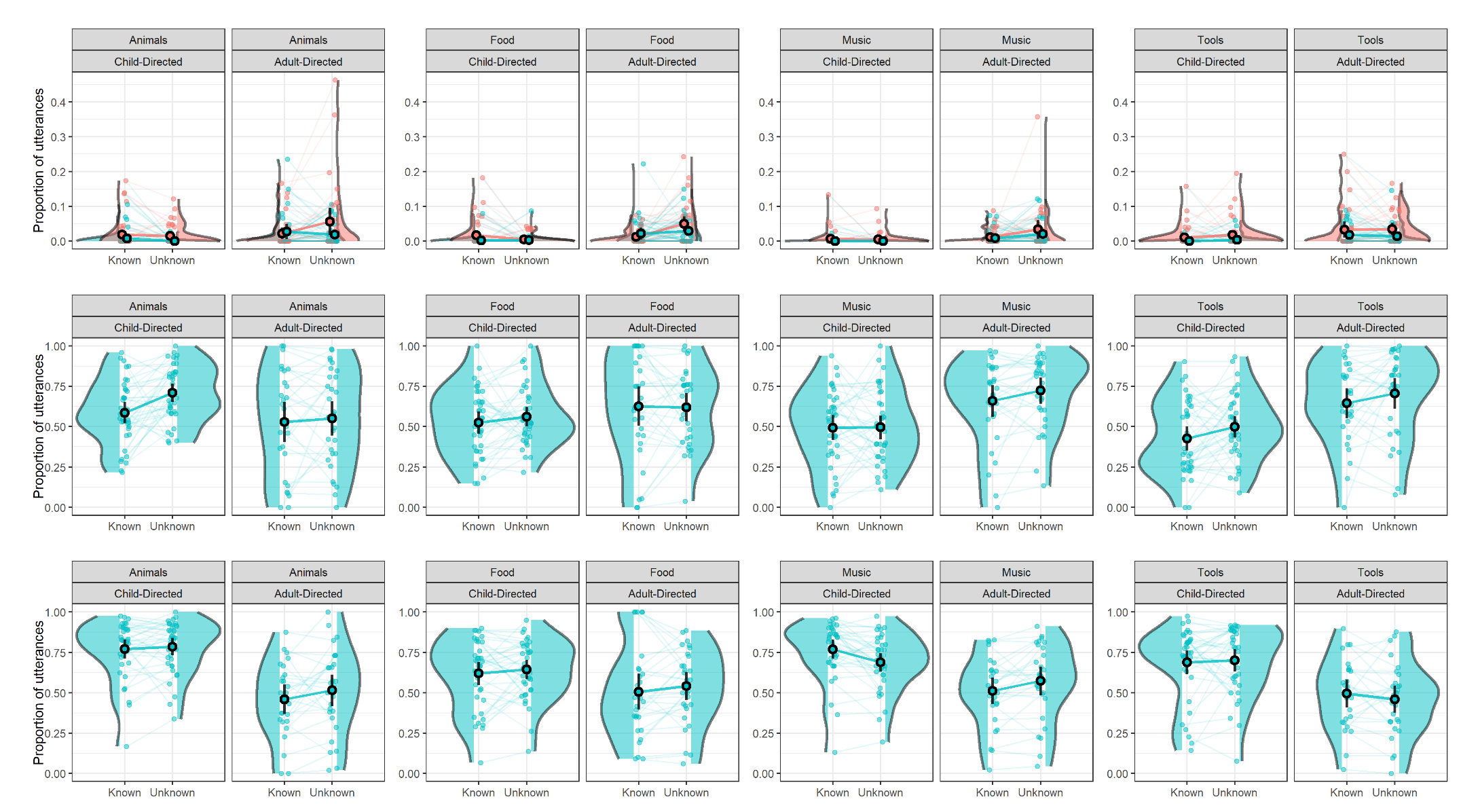


**7d**

**8**

**9**
